# Advancing Therapeutic Targets in IBD: Emerging Goals and Precision Medicine Approaches

**DOI:** 10.3390/ph18010078

**Published:** 2025-01-10

**Authors:** Lucia Centanni, Clelia Cicerone, Fabrizio Fanizzi, Ferdinando D’Amico, Federica Furfaro, Alessandra Zilli, Tommaso Lorenzo Parigi, Laurent Peyrin-Biroulet, Silvio Danese, Mariangela Allocca

**Affiliations:** 1 Gastroenterology and Endoscopy, IRCCS Hospital San Raffaele, University Vita-Salute San Raffaele, 20132 Milan, Italy; 2 Department of Gastroenterology, INFINY Institute, INSERM NGERE, CHRU de Nancy, Université de Lorraine, F-54500 Vandœuvre-lès-Nancy, France

**Keywords:** new therapeutic targets, clinical remission, endoscopic remission, histologic remission, transmural remission, deep remission

## Abstract

Inflammatory bowel diseases (IBD) including Crohn’s disease (CD) and ulcerative colitis (UC) are chronic, relapsing conditions characterized by dysregulated immune responses and persistent intestinal inflammation. This review aims to examine new potential therapeutic targets in IBD starting from the STRIDE-II statements. Key targets now include clinical remission, endoscopic remission, and biomarker normalization (such as C-reactive protein and fecal calprotectin). Moreover, histologic remission, transmural remission, and in the future molecular targets are emerging as important indicators of sustained disease control. The treatment goals for inflammatory bowel disease are varied: to relieve symptoms, prevent permanent intestinal damage, promote inflammation remission, and minimize complications. Consequently, the therapeutic targets have evolved to become broader and more ambitious. Integrating these advanced therapeutic targets has the potential to redefine IBD management by promoting deeper disease control and improved patient outcomes. Further research is essential to validate these strategies and optimize their clinical implementation.

## 1. Introduction

Inflammatory bowel disease (IBD), including ulcerative colitis (UC) and Crohn’s disease (CD), is a chronic and relapsing inflammatory disorder of the gastrointestinal tract [1]. Effective disease management aims not only to relieve symptoms, but also to achieve deeper therapeutic targets, which ultimately influence long-term patient outcomes [2]. Traditionally, treatment goals following the treat-to-target approach (T2T) focused on clinical remission (the absence of symptoms) and endoscopic remission (resolution of visible mucosal inflammation) [3].

The introduction of the STRIDE-II guidelines in 2021 marked a significant shift in IBD management, broadening therapeutic targets to encompass histologic remission (remission at the microscopic tissue level) and transmural remission (in CD, reflecting full-thickness remission of the bowel wall) [3]. These targets provide a more comprehensive view of disease control, as clinical remission alone often fails to predict long-term remission or prevent complications [3]. Additionally, biomarker normalization (such as C-reactive protein (CRP) and fecal calprotectin (FC)) has emerged as an intermediate treatment target since its being a surrogate marker for ongoing inflammation and disease activity [4]. Recent studies indicate that achieving these expanded therapeutic targets (histologic remission and transmural remission) correlates with improved outcomes, including reduced hospitalization, lower surgical rates, and prolonged remission [5]. This review explores the latest evidence on new therapeutic targets.

## 2. Crohn’s Disease: Current Therapeutic Targets

The STRIDE-II initiative outlines key targets for managing Crohn’s disease, with a primary emphasis on long-term endoscopic remission (ER), as it is linked to reduced bowel damage and better overall outcomes [3]. ER remains the central goal, as ongoing mucosal inflammation even in the absence of clinical symptoms is associated with future complications [6]. Clinical symptoms alone are insufficient to assess inflammation, so objective measures like FC and CRP, along with non-invasive tools like intestinal ultrasound (IUS), provide effective and cost-efficient monitoring alternatives [4]. The comprehensive, multi-target approach advocated by STRIDE-II has been already considered in studies like CALM and STORI, which support combining clinical, biochemical, and endoscopic data to achieve better outcomes [7,8]. STRIDE-II, following this approach, also emphasizes the importance of personalized treatments for CD. Emerging targets, including transmural remission (TR) and histologic remission (HR), hold promise but require clearer definitions and further study for clinical integration [4].

### 2.1. Clinical Remission

The Crohn’s Disease Activity Index (CDAI) and the Harvey–Bradshaw Index (HBI) are two commonly used tools to assess disease activity in CD [9]. The CDAI is comprehensive, incorporating eight parameters like stool frequency, abdominal pain, general well-being, extra-intestinal complications, and laboratory results. It offers a detailed assessment but requires tracking symptoms over a week and complex calculations, making it more suitable for research than daily clinical practice [10]. In contrast, the HBI is simpler, focusing on five main parameters: general well-being, abdominal pain, number of stools per day, presence of complications, and abdominal mass. This makes it more practical and widely used in routine clinical settings [9]. While the CDAI provides a broader evaluation, the HBI offers a quicker, yet reliable snapshot of disease activity [9,10]. The CDAI and HBI are commonly used to assess symptom severity; however, these indices may not always accurately reflect underlying inflammation, as symptoms can persist despite minimal disease activity or in the absence of disease inflammation [10,11]. This highlights the importance of patient-reported outcome measures (PROMs), which capture the patient’s perspective on symptom burden and quality of life. PROMs, such as the CD-PRO2, are valuable for tracking daily symptoms like stool frequency and abdominal pain, offering a more comprehensive understanding of how patients perceive disease control and helping to bridge the gap between physician assessments and patient experiences [12,13].

### 2.2. Biomarker Remission

In CD, FC and CRP are the most widely utilized biomarkers to assess inflammatory activity. FC, a non-invasive fecal marker, is sensitive to mucosal inflammation and useful for distinguishing active from inactive disease, especially in colonic involvement [14,15]. One of the abilities of FC is its ability to act as an early indicator of treatment response, enabling clinicians to identify subclinical inflammation before symptoms emerge [16]. Moreover, FC has shown high diagnostic accuracy, with sensitivities up to 92% and specificities up to 82% depending on the cut-off value, allowing for precise monitoring of disease activity [14], as well as decreasing FC levels after initiating therapy can predict sustained remission and a lower risk of relapse. For example, a reduction in FC by at least 50% at week 12 after anti-TNF therapy has been associated with corticosteroid-free remission at 1 year [17]. However, FC can yield false positives in other inflammatory conditions, limiting specificity [18]. CRP, a serum marker produced in response to IL-6 and TNF-α, is widely used to monitor disease activity, particularly in CD. However, CRP is neither bowel- nor disease-specific, and approximately 20% of patients with active ileal CD may have normal CRP levels [17]. Despite these limitations, timely CRP measurement during therapy has proven predictive of treatment response, with normalization within 8–14 weeks after initiating anti-TNF therapies strongly correlating with remission at one year and the suggestion that a 60% decrease in CRP by week 14 is associated with a higher likelihood of sustained remission [17,19,20]. Combining biomarkers like FC and CRP enhances the predictive accuracy of disease outcomes. For example, the CALM trial demonstrated that integrating both markers with clinical symptoms improves the prediction of endoscopic remission compared to symptoms alone [17]. Furthermore, a decline in FC levels early after therapy initiation predicts long-term remission and endoscopic healing in both CD and UC [16] and a decrease in FC and CRP levels after initiating therapy can predict sustained remission and a lower risk of relapse [21,22]. The variability in calprotectin results across different assays and the lack of standardization can complicate its interpretation, highlighting the need for cautious evaluation, especially in borderline cases [16]. STRIDE-II guidelines recommend an FC level below 150 µg/g to indicate ER, though values between 100–250 µg/g are often treated cautiously as an intermediate range [23]. Emerging evidence suggests that lower FC thresholds (<150 µg/g) might better predict histologic or transmural remission, underscoring the need for individualized cut-offs tailored to disease location and severity [17]. CRP normalization is also a target for remission [19]. However, defining a “normal” CRP level remains complex due to individual variability and the absence of standardized thresholds. While levels below 10 mg/L are generally considered normal, lower thresholds (<5 mg/L, <3 mg/L, <1 mg/L) may more accurately reflect minimal inflammatory activity. Despite this, some patients with clinically active disease may still exhibit normal CRP levels, making it challenging to distinguish mild inflammation from functional symptoms. These discrepancies emphasize the need to interpret CRP levels in conjunction with clinical assessments and other biomarkers to achieve a comprehensive evaluation of disease activity [20,24].

Persistent elevation of CRP or FC during treatment should prompt clinicians to re-evaluate therapeutic strategies, as these biomarkers are indicative of ongoing inflammation even in asymptomatic patients [25].

The erythrocyte sedimentation rate (ESR) serves as an additional inflammatory marker, though it is less specific and often slower to respond to changes in disease activity than CRP. ESR may be useful in conjunction with CRP and FC for a broader view of inflammatory status, particularly in cases where CRP response is minimal, though it can be influenced by factors like anemia and pregnancy [25].

Combining FC and CRP with clinical assessments provides a more thorough approach to evaluating CD activity, supporting current multi-targeted management strategies aimed at optimizing treatment and preventing disease progression.

### 2.3. Endoscopic Remission

Endoscopic remission is a cornerstone in the long-term management of CD, emphasizing the resolution of mucosal inflammation over mere symptom control. Achieving this target correlates with improved outcomes, including sustained remission, reduced corticosteroid dependence, lower rates of hospitalizations, and fewer surgical interventions. STRIDE-II guidelines identify ER as a critical goal, with recommendations for assessment within 6–9 months of therapy initiation [4].

Validated scoring systems such as the Crohn’s Disease Endoscopic Index of Severity (CDEIS, range: 0–44 points) and the Simple Endoscopic Score for Crohn’s Disease (SES-CD, range: 0–60 points) provide a structured approach to quantify inflammation and ulceration, hallmarks of CD pathology. Endoscopic response is typically defined as a ≥50% reduction in SES-CD or a ≥5-point decrease in CDEIS, while ER corresponds to scores of SES-CD < 3 or CDEIS < 3 [26]. Mucosal remission, the ultimate goal, is marked by the complete absence of ulcers [26,27].

This therapeutic focus stems from robust evidence linking mucosal remission to durable disease control. For instance, early achievement of this target has been associated with long-term remission, fewer complications, and improved quality of life. Trials like SONIC have highlighted the importance of mucosal remission in reducing corticosteroid use and preventing relapse [4,28,29,30,31]. However, endoscopic evaluation with ileocolonoscopy is invasive, costly, and time-consuming. Additionally, ileocolonoscopy has certain limitations, including restricted access to the proximal small bowel and the possibility of underestimating disease activity in the presence of strictures, which are often missed in about 50% of small bowel cases [32]. This underscores the gap between ER and TR, with the latter reflecting the full resolution of inflammation across all bowel layers [33]. Given these limitations, combining endoscopic evaluation with non-invasive monitoring tools provides a more comprehensive and accessible approach [3,34].

## 3. Crohn’s Disease: Emerging Therapeutic Targets

### 3.1. Histologic Remission

Histologic remission has garnered increasing interest as a potential marker of deeper disease control, aligning with evidence that deeper remission correlates with better long-term outcomes in CD [35].

Despite its potential benefits, HR is not currently a formal treatment target in CD, as reflected in the STRIDE-II recommendations and in the ESGE guidelines on endoscopic tissue sampling in CD. ESGE guidelines caution against routine biopsies for assessing histologic disease activity in known CD, highlighting significant limitations. These include the lack of sufficient validation for histologic scores, the variable correlation with endoscopic disease activity, and the sampling bias inherent in biopsies due to the patchy distribution of CD. Additionally, mucosal biopsies may not accurately represent the transmural nature of the disease, and access to the most inflamed areas may be restricted by strictures, further complicating the clinical utility of histologic assessment. These challenges underscore the limited impact of histologic findings on treatment decisions and question the routine incorporation of HR as a clinical endpoint [36,37]. Such limitations highlight the complexity of incorporating histologic endpoints into routine clinical management [3,4]. Key challenges include the absence of standardized, validated scoring systems and insufficient evidence to justify escalating immunosuppressive therapy solely to achieve HR. Furthermore, current therapeutic options, including biologics, have shown limited effectiveness in consistently inducing HR in CD [3]. HR is associated with lower rates of clinical relapse and complications in CD, as evidenced by studies showing that patients with HR have better long-term outcomes [36]; however, only a small proportion of patients achieve this endpoint even after attaining clinical and ER [38].

### 3.2. Transmural Remission

While TR is not yet a formal treatment target also of the limitations of available therapies, its role as an adjunctive measure is increasingly recognized, as endorsed by the IOIBD Delphi consensus [3].

CD is a transmural condition, which makes TR an important factor to consider, in addition to mucosal remission. Recent research shows that TR is linked to better clinical outcomes, such as higher rates of steroid-free remission, fewer hospitalizations, and reduced need for surgical interventions, when compared to MR alone. Imaging tools like intestinal ultrasound (IUS), magnetic resonance enterography (MRE), and computed tomography enterography (CTE) are crucial for evaluating TR, as they offer non-invasive insights into disease activity and complications, such as bowel wall thickness, vascularization, and mesenteric inflammation, all of which are correlated with prognosis [34,39]. In this context, the Bowel Ultrasound Score (BUSS), a quantitative IUS-based measure that combines bowel wall thickness (BWT) and bowel wall flow (BWF), has been demonstrated to predict disease outcomes in CD patients over a 12-month period [40].

The relationship between TR and ER observed in various studies underscores the potential of radiologic techniques as alternatives to colonoscopy for monitoring disease progression [32]. For instance, a study by Oh et al. showed that patients who achieved both ER and radiologic remission had significantly better survival rates free from major adverse outcomes compared to those who achieved only ER, only radiologic remission, or neither (*p* < 0.001). This highlights the complementary roles of radiologic evaluation and endoscopy in managing CD [41]. In addition, it has been demonstrated that changes in BUSS over time have the ability to predict therapeutic response and endoscopic response (a reduction of at least 1.2 points), in particular identifying endoscopic response with 80% accuracy and ER with 78% accuracy; these findings confirm the utility of IUS in detecting changes in lesion severity over time [42]. Another study showed that, at week 12, ultrasound remission identified through IUS predicts long-term ER in patients undergoing biologic therapy, emphasizing its potential as a non-invasive surrogate for TR assessment [43]. Despite these advancements, TR is not yet considered a primary treatment target. However, incorporating TR as a complementary endpoint alongside mucosal remission could enhance therapeutic decision-making and long-term monitoring [39]. TR can be assessed using various techniques: MRE offers detailed visualization, making it ideal for complex cases, CTE is preferred in emergencies but involves radiation exposure, and IUS is particularly effective for routine monitoring of disease activity, providing additional prognostic insights by evaluating vascularization and bowel wall inflammation during treatment. Moreover, IUS is non-invasive, cost-effective, and allows for dynamic assessments, making it an essential tool for adjusting therapy [44].

## 4. Ulcerative Colitis: Current Therapeutic Targets

In recent years, treatment goals for UC have evolved significantly, marking a shift towards more personalized and proactive management. The treat-to-target (T2T) strategy has become a cornerstone, focusing on early and continuous monitoring to tailor treatment according to specific, well-defined goals [45]. This approach underscores the importance of a multidisciplinary team to optimize therapeutic decisions and improve patients’ quality of life [45]. Clinical remission, defined by the resolution of rectal bleeding and the normalization of stool frequency, remains the primary treatment objective. The secondary objective is that ER is a critical milestone strongly associated with reduced relapse rates and long-term disease control [3]. Biomarkers, such as CRP and FC, offer convenient, non-invasive methods to track inflammatory activity and guide treatment adjustments [4]. While HR is not yet universally recognized as a routine endpoint, it is gaining recognition for its potential to signify deeper disease remission, as histologic inflammation can persist even after mucosal remission [3]. In recent years, “disease clearance” has been proposed as a composite target, encompassing deep clinical, endoscopic, and HR [46].

### 4.1. Clinical Remission

In UC, clinical symptoms are closely correlated with the severity of endoscopic inflammation, making clinical remission a crucial therapeutic goal [39]. Clinical remission is commonly assessed using the Mayo score, which is a standard outcome measure in clinical trials. Complete clinical remission is defined by normal stool frequency, absence of rectal bleeding, and no abdominal pain, and it is strongly associated with ER in the majority of patients [47]. The PRO2 score, derived from the stool frequency and rectal bleeding components of the Mayo score, is a valuable tool for assessing symptoms in UC. These two parameters are key indicators of disease activity, and the moderate-to-high correlation between PRO2 and ER emphasizes the need for additional inflammation measures to ensure accurate disease monitoring. Clinical remission in UC is typically defined as achieving a PRO2 score of 0 for both rectal bleeding and stool frequency, or a partial Mayo score below 3, with no individual subscore exceeding 1 [47]. A significant number of patients, particularly those with active rectal inflammation, also report symptoms such as bowel urgency, incontinence, tenesmus, and a persistent feeling of incomplete evacuation [48].

While normalizing bowel habits, resolving rectal bleeding, and controlling urgency are key indicators of symptomatic remission, these factors alone may not fully capture a patient’s sense of recovery, as other aspects -such as overall well-being, fatigue, and work productivity- significantly affect their quality of life [7]. Anyway, when these targets are not achieved, therapeutic adjustment is strongly suggested to optimize disease control and prevent progression.

### 4.2. Biomarker Remission

The STRIDE-II recommendations identify the normalization of CRP/ESR levels (below the upper limit of normal) and FC (to 100–250 mg/g) as intermediate treatment targets in UC [3]. FC is more sensitive than CRP and ESR [49]. Though FC can show false positives in some conditions, limiting specificity [17], its superior correlation with endoscopic activity compared to CRP makes it an indispensable tool in UC, where it demonstrates better performance than in CD [25]. FC is a predictor for clinical relapse, sustained clinical response, loss of response to maintenance therapy after induction, ER, and histological mucosal remission [50,51,52,53,54]. Better correlating with endoscopic disease activity than symptom-based indices, particularly in UC, its sensitivity and specificity make it a reliable surrogate marker for monitoring mucosal healing [17,25]. For this reason, its assessment becomes increasingly important in the clinical setting, helping physicians monitor treatment response [55]. However, changes in FC levels may not always accurately reflect shifts in disease activity or guide therapeutic adjustments when a patient is in clinical remission. In these cases, variability in FC levels often lacks clinical significance, potentially leading to an overestimation of disease activity [56,57].

FC levels can be influenced by various factors that limit their reliability in assessing disease activity. These include intestinal infections, use of nonsteroidal anti-inflammatory drugs, recent gastrointestinal bleeding, and non-IBD conditions such as diverticulitis or colorectal cancer. Additionally, variations in stool consistency and sampling methods can impact FC measurements. These limitations confirm that while FC is a useful non-invasive marker, its interpretation should be contextualized within the clinical and diagnostic setting [58,59].

### 4.3. Endoscopic Remission

Endoscopic remission in UC is a critical therapeutic goal associated with improved long-term outcomes, including reduced complications, hospitalizations, surgical interventions, and colorectal cancer. This reflects a broader evolution in UC treatment goals, shifting from mere symptom control to prioritizing mucosal remission and preventing severe complications such as disability, colectomy, and cancer [49,60].

In UC, ER is commonly evaluated using validated scoring systems. The Mayo Endoscopic Subscore (MES) and the Ulcerative Colitis Endoscopic Index of Severity (UCEIS) are the most frequently studied tools. ER is typically defined as achieving an MES ≤ 1, which evaluates the severity of inflammation based on vascular pattern, friability, and ulceration, including mild residual inflammation. However, achieving complete ER, characterized by an MES of 0 (no visible inflammation), is linked to better long-term clinical outcomes, including reduced relapse rates, improved quality of life, and improved PRO scores [61,62]. A meta-analysis of 17 studies involving 2608 patients with ulcerative colitis (UC) in clinical remission demonstrated that patients with MES 0 had a 52% lower risk of clinical relapse over 12 months compared to those with MES 1 (RR 0.48; 95% CI 0.37–0.62). The annual risk of clinical relapse was approximately 13.7% for patients with MES 0, compared to 28.7% for MES 1 [63].

The UCEIS is a more detailed scoring system ranging from 0 to 8 that evaluates vascular pattern, bleeding, and erosion/ulceration; this scoring system provides a more granular assessment of inflammation and correlates well with histological findings, offering greater objectivity, especially in clinical trials and research [64]. Moreover, monitoring minimal or absent inflammation through endoscopic evaluation can help lower the likelihood of malignancy and this is extremely relevant since intestinal inflammation is a recognized independent risk factor for the development of colon cancer associated with colitis [65].

The optimal timing for evaluating ER following treatment initiation in UC has not been conclusively defined. Current recommendations suggest performing an endoscopic assessment within 3–6 months of starting therapy in UC patients with symptoms [49]. This timeframe allows for informed decisions regarding potential treatment adjustments. Studies employing proactive monitoring strategies, including mucosal evaluation within six months of initiating biologic therapy, have demonstrated benefits such as reduced reliance on corticosteroids in UC patients [66]. Nevertheless, the frequency of monitoring should be tailored to disease severity and the patient’s clinical history [62].

Mucosal remission is also critically important for UC outcomes, as it plays a key role in lowering the risk of dysplasia and colorectal cancer [67].

Recent advancements in endoscopic techniques have greatly enhanced the detection of mucosal healing and dysplasia in UC patients, a critical factor in preventing progression to colorectal cancer. Chromoendoscopy, especially high-definition virtual chromoendoscopy (VCE), has become crucial for enhancing endoscopic evaluation, improving the accuracy of histological predictions, and monitoring dysplasia [67]. Artificial intelligence (AI) is emerging as a game-changer in endoscopic practice, with systems like CADe Discovery™ showing performance equal to or surpassing traditional methods, significantly enhancing dysplasia detection. When combined with chromoendoscopy, AI offers real-time analysis, enabling the identification of subtle mucosal abnormalities that might otherwise be missed [68]. Although confocal laser endomicroscopy (CLE) and endocytoscopy are still being assessed for UC-specific applications, they are valued for their ability to deliver high-resolution, detailed mucosal imaging. These techniques show promise in detecting subtle dysplastic changes, but additional research is necessary to establish their role in routine surveillance [69,70,71].

## 5. Ulcerative Colitis: Emerging Therapeutic Targets

### 5.1. Histologic Remission and Disease Clearance

Histologic assessment plays a critical role in managing UC, offering insights into tissue inflammation, damage, and remission that complement endoscopic findings. HR correlates with improved long-term outcomes, including reduced relapse rates, steroid dependency, and hospitalizations [72,73,74,75,76] highlighting its importance as a treatment target [72,77,78]. Achieving ER alone is insufficient, as patients may still experience histological disease activity, leading to worse prognoses [46].

There are several endoscopic scores used to document HR. The most commonly used score is the Nancy score, with a score of Nancy < 1 defined as HR. Recently it has been proposed the “PICaSSO Histologic Remission Index” a novel score based on the presence or absence of neutrophils (yes/no), offering excellent diagnostic accuracy. It demonstrates the strongest correlation with endoscopic activity among various histological scores, minimal inter-rater variability, and robust prediction of long-term clinical outcomes [79,80].

Persistent histologic activity, such as basal plasmacytosis and surface irregularities, even in the presence of mucosal remission, is associated with higher relapse risks and colorectal neoplasia, emphasizing the need for biopsy sampling in routine evaluations, which conversely is not recommended to assess disease activity in CD [37,81,82,83]. Though HR is not yet formally recognized as a standard target, the European Crohn’s and Colitis Organization (ECCO) proposes the definition of HR at least as the absence of intraepithelial neutrophils, erosion, and ulceration as a minimum requirement [83].

Emerging technologies, including advanced imaging and artificial intelligence, promise to enhance histological evaluations, enabling more precise and tailored treatment strategies. However, the integration of HR into routine practice requires further research to establish standardized definitions, protocols, and its broader clinical relevance [84]. Ultimately, if clinical remission in UC has been a significant therapeutic target and it may not accurately reflect the inflammatory burden, on the other side HR should be considered as a potential new formal target for better disease management [85].

However, achieving HR alone may still not be sufficient for comprehensive disease management, as UC involves a complex interplay of clinical, endoscopic, and histological factors that collectively influence prognosis. This recognition has shifted the focus toward a more holistic treatment goal: disease clearance (DC) [86].

By combining clinical symptom resolution, ER, and HR into a single objective, DC provides a multidimensional approach to disease control, with improved patient outcomes. By encompassing clinical symptoms, endoscopic findings, and histological results, DC provides a holistic view of disease status and holds prognostic value by correlating with better long-term outcomes and reduced risks of complications [87]. An analysis of 494 patients with UC revealed that, at baseline, DC was observed in 109 patients (22.1%). Over a median follow-up of 24 months, patients with DC demonstrated significantly better outcomes, including a lower risk of UC-related hospitalization compared to the control group (5.5% vs. 23.1%; *p* < 0.001) at the last observation. Additionally, the rate of surgeries was notably lower among patients with baseline DC compared to those without (1.8% vs. 10.9%; *p* = 0.003) with Kaplan–Meier analyses further confirming DC at baseline as associated with significantly reduced risk of both hospitalization (log-rank *p* < 0.0001) and surgery (log-rank *p* < 0.00095) [88].

The VERDICT trial is the first to evaluate whether a treatment strategy targeting DC outperforms symptom control alone or combined with ER. This ongoing trial aims to guide UC management by assessing the impact of each component of DC and serving as a foundation for future disease-modification trials. The interim analysis of the VERDICT trial offers compelling evidence regarding the effectiveness of vedolizumab in achieving DC, defined as corticosteroid-free symptomatic remission, endoscopic improvement, and HR in patients with moderate-to-severe UC. After 16 weeks of treatment, 41% of patients in the target group reached this stringent remission target, demonstrating the feasibility of achieving comprehensive disease control with this protocol. Notably, bionaive patients exhibited higher success rates (41%) compared to bio-exposed patients (35%). The study enrolled 553 patients, with 253 assigned to the target group, and the treatment protocol featured early initiation of vedolizumab without dose escalation before week 16, underscoring the importance of timely intervention in achieving DC. These findings have significant implications, demonstrating that corticosteroid-free DC may serve as a marker for potential disease modification. Further research is warranted to validate these outcomes and assess the long-term benefits of this treatment strategy, providing valuable insights into optimizing UC management and improving patient quality of life [89].

DC is a challenging target that fewer than 20% of patients typically reach within one year of treatment with existing therapies, highlighting room for improvement [87]. Ongoing research, including efforts by the International Organization for the Study of Inflammatory Bowel Diseases, seeks to refine the definition and standardize criteria for DC [90]. Additionally, future strategies may target molecular remission, further expanding the scope of DC. The need for validated definitions and the limited proportion of patients currently achieving this ambitious composite outcome remains a topic for current and future research [87].

### 5.2. Emerging Ultrasound Markers of Disease Activity

IUS is a non-invasive tool used to assess disease activity in UC. It facilitates quick treatment decisions, potentially modifying disease progression and improving patient compliance, making it a valuable option in monitoring UC [91].

The primary parameter evaluated in IUS is bowel wall thickness (BWT), measured across all colonic segments, which reflects the degree of mucosal inflammation [92]. Alongside BWT, the color Doppler signal (CDS) is another reliable indicator of intestinal inflammation, with vascular signals graded semi-quantitatively using the Limberg score [93]. This score ranges from 0 (normal wall thickness without vascular spots) to 4 (extensive vascularization extending beyond the bowel wall into surrounding tissues). Another key parameter, bowel wall stratification (BWS), describes the loss of differentiation between bowel wall layers, indicating mucosal damage such as erosions or deep ulcers. Indirect signs, such as swollen lymph nodes or hypoechoic peri-intestinal mesenteric fat (i-fat), can help detect milder inflammatory activity [92,94].

A 2003 study by Parente et al. identified BWT as the primary ultrasound marker of disease activity, achieving 87% sensitivity in detecting inflammation in the descending and sigmoid colon, with colonoscopy as the reference standard [95]. More recently, significant correlations have been demonstrated between IUS markers (BWT, CDS, mesenteric lymph node enlargement, and hypoechoic fat) and endoscopic activity (Mayo score > 2). Multivariable analysis revealed that a 1 mm increase in BWT (odds ratio [OR]: 4.05) and CDS (OR: 7.99) were predictors of endoscopic inflammation. These findings highlight the utility of IUS in assessing disease activity and predicting mucosal inflammation in UC [96]. More recently, the Milan Ultrasound Criteria (MUC), the first rigorous scoring system for assessing UC activity via IUS, was introduced. The MUC formula is defined as 1.4 × BWT (mm) + 2 × CDS (1 if present and 0 if absent). Receiver operating characteristic analysis in the study determined an MUC > 6.2 as the optimal cut-off for identifying disease activity: it predicts disease course in UC and MUC ≤ 6.2 may be the new treatment target to achieve reduce the risk of worse outcomes [97].

The evaluation of rectal inflammation in UC through IUS remains a significant challenge due to anatomical complexities and interference from nearby structures, such as the bladder [92]. IUS has shown lower accuracy for rectal activity compared to other colonic segments, with sensitivity considerably reduced [98] Transperineal ultrasound (TPUS) has recently emerged as a promising tool for rectal assessment, showing superior correlations between markers like bowel wall thickness (BWT) and color Doppler signal (CDS) with endoscopic and histological findings compared to trans-abdominal IUS [99]. Pilot studies in UC patients have highlighted TPUS’s high sensitivity and diagnostic potential, suggesting it could play a pivotal role also in histological remission assessment [99], though further research is needed to confirm its utility and feasibility in routine clinical practice.

Anyway, a superior predictive value was found for transmural vs. endoscopic severity for colectomy risk in UC patients with MUC emerging as the only independent predictor of this outcome in multivariable analysis. Its robust performance supports its potential as a valuable tool in clinical decision-making for UC [100].

## 6. Molecular Targets in IBD

Biomarkers, as non-invasive and reproducible tools, play a critical role in managing IBD. The search for new biomarkers is intensifying, with a significant focus on proteomics, genetics, and metabolomics. Since no single biomarker is sufficient for all stages of IBD management, developing personalized biomarker panels is essential. However, challenges remain, including standardization of tests, data interpretation, and ethical or logistical issues associated with personalized medicine [101].

Proteomics, which examines protein expression and post-translational modifications, holds great promise for understanding IBD pathogenesis and identifying novel biomarkers. Techniques like mass spectrometry (LC–ESI-MS/MS) have advanced research, aiding in distinguishing IBD from other intestinal diseases, differentiating UC and CD, and predicting therapeutic responses and neoplastic transformations. Preliminary studies have revealed early protein expression changes associated with cancer risk in IBD patients, but larger-scale validation is needed. Despite hurdles such as high costs, technical complexity, and data integration challenges, proteomics remains a promising avenue for advancing personalized medicine in IBD care [102,103].

Genetic factors play a significant role in IBD, influencing immune responses to intestinal microbiota. Genome-wide association studies (GWASs) have identified around 240 gene loci associated with IBD susceptibility, aiding in understanding disease mechanisms and advancing biomarker discovery [104].

Gene profiling has revealed distinct panels differentiating IBD patients from healthy individuals, as well as specific patterns distinguishing UC and CD or active and remission phases in CD [105,106,107,108].

A positive family history is one of the strongest risk factors for IBD, with first-degree relatives showing significantly higher incidence rates, especially in individuals under 20 years old. This highlights the potential of early genetic screening in high-risk populations. Furthermore, genetic variations like NOD2 mutations in CD have been linked to aggressive treatment needs, such as anti-TNF therapy. Studies have also identified specific gene markers, including TLR2 and others, that predict anti-TNF response, particularly in pediatric patients [107,109,110,111].

While these findings hold promise for personalized medicine, challenges remain in integrating genetic data into clinical practice due to the complex interaction of genes and environmental factors. Future research should focus on validating genetic biomarkers in diverse populations and combining genetic insights with other clinical data for improved diagnosis and treatment strategies [101].

Furthermore, epigenetics holds great promise for advancing IBD research and identifying biomarkers for disease progression and treatment [112]. Epigenetics involves studying heritable changes in genome function that occur without altering the DNA sequence. Key mechanisms include DNA methylation, histone acetylation, RNA interference, and nucleosome positioning. These processes, influenced by gene-environment interactions, play a significant role in IBD pathogenesis and biomarker discovery [112]. Research highlights the importance of DNA methylation in IBD, with studies identifying differential methylation patterns in UC and CD compared to healthy controls. Specific genes, such as THRAP2, FANCC, GBGT1, DOK2, TNFSF4, TNFSF12, and FUT7, show altered methylation that may contribute to disease progression. IBD is strongly associated with an elevated risk of colorectal cancer, driven by chronic inflammation and epigenetic changes that promote metabolic reprogramming and uncontrolled cell division. Biomarkers such as linoleic acid, metabolic gene expression, and microRNAs (e.g., miR-31, miR-155, miR-223) are being explored for early CRC detection and risk assessment in IBD patients [113,114]. Considering the importance of host-microbiota interactions in the context of IBD, these relationships play a pivotal role in shaping immune responses and metabolic pathways. It has been recently demonstrated that specific colonic microbial taxa, such as Pacebacteria, Streptophyta, and Aerophobetes, interact with host pathways like PI3K-Akt signaling. These interactions influence immune responses and enhance intestinal metabolism, providing valuable insights into potential microbiota-mediated therapeutic strategies. Understanding the dual regulation of immune and metabolic pathways by gut microbiota and host genes underscores the importance of these interactions in advancing IBD management and these findings open avenues for further exploration of microbiome-targeted strategies that could complement existing therapies [115].

Despite significant advancements, challenges remain in leveraging molecular research, including epigenetics and metabolomics, to better understand IBD. Epigenetic patterns vary across cell types, complicating bulk tissue analysis, and it is often unclear whether these changes are causes or consequences of IBD, as they can fluctuate with time and environmental factors. Current profiling methods are costly and labor-intensive, and functional validation of identified epigenetic alterations requires extensive research. Similarly, metabolomics has revealed promising biomarkers reflecting disease activity and progression, highlighting the intricate interplay between gut microbiota and host metabolism. These insights underscore the roles of specific metabolites and microbial-derived molecules in distinguishing IBD from healthy states and advancing our understanding of disease mechanisms [116,117,118,119].

To fully harness these tools, future priorities include developing single-cell epigenomic techniques to address cellular heterogeneity, integrating epigenetic and metabolomic data with other omics for a comprehensive view of IBD, and conducting longitudinal studies to track dynamic changes over time. Moreover, more efficient and cost-effective profiling methods, along with functional studies, are essential to clarify the roles of epigenetic modifications and metabolomic alterations. By combining insights from microbiota analysis and molecular profiling, researchers are uncovering novel biomarkers and therapeutic targets, paving the way for precision medicine and improved clinical management of IBD [101,116].

Advancements in biomarker research, combined with artificial intelligence (AI) and machine learning (ML) may revolutionize IBD management. By integrating complex datasets from various sources, AI uncovers patterns that enhance understanding of IBD’s etiology and support the development of personalized therapies. AI applications include accurate diagnosis, monitoring disease activity, predicting treatment responses, and forecasting complications, while also improving cancer surveillance in high-risk patients. Endoscopic tools powered by AI, such as convolutional neural networks, show superior accuracy in lesion detection and disease assessment. However, challenges like data bias, heterogeneity, and the need for methodological standardization remain. Collaborative efforts and robust validation are crucial to fully integrate AI into clinical practice, advancing precision medicine and improving patient outcomes [101,120].

## 7. Discussion and Future Prospectives

The treat-to-target (T2T) strategy has revolutionized IBD management by emphasizing clinical remission, ER, and biomarker normalization. While these targets have significantly improved patient outcomes, they often overlook deeper aspects of disease activity. Emerging goals, such as TR in CD and HR in UC, aim to address these gaps, offering a more comprehensive approach to long-term disease control. Furthermore, the integration of molecular targets promises to advance personalized medicine in IBD (Table 1).

In CD, TR is an evolving target that signifies a complete resolution of inflammation across all bowel layers, addressing disease processes not captured by ER alone, such as abscesses, fistulas, and extraluminal inflammation. Despite its potential, defining TR consistently and understanding its role in treatment adjustments remain challenges. Real-world studies and the refinement of imaging techniques are crucial to establishing TR as a standard therapeutic goal, particularly since current treatments do not consistently achieve it.

In UC, HR is increasingly recognized as a marker of deeper disease control and is associated with better long-term outcomes. Achieving HR may become a key target for long-term disease management. The concept of disease clearance has gained recognition, as shown by the VERDICT trial, but incorporating HR into routine practice faces challenges, such as variability in biopsy techniques, lack of standardized scoring systems, and difficulty achieving this goal, particularly in severe disease phenotypes. Addressing these issues could redefine treatment goals, ensuring more precise disease control.

**Figure 1:**
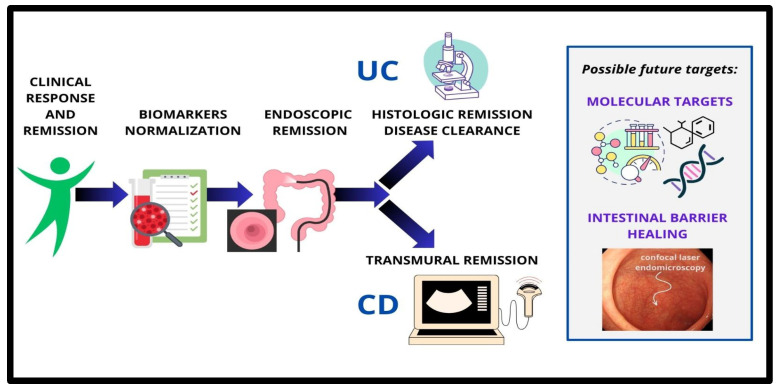
Evolution of specific targets in the management of IBD.

Beyond HR, barrier remission, assessed through confocal laser endomicroscopy (CLE), has emerged as a promising new target [121]. CLE has shown a reduced risk of disease progression in clinically remitted IBD patients and has superior predictive performance compared to ER and HR. CLE is able to identify local barrier dysfunctions in IBD patients, even in the absence of macroscopic inflammation [122]. Evidence from CLE studies suggests that barrier remission is strongly linked to reduced risks of disease flares, hospitalizations, and surgeries in both UC and CD. Additionally, CLE imaging has demonstrated high accuracy in predicting long-term outcomes, particularly when assessing the terminal ileum in CD patients, highlighting the potential for barrier remission to become a critical therapeutic endpoint in the future [122,123]. (Figure 1).

In the context of IBD-associated colorectal cancer, recent advancements in computational technologies, particularly machine learning, have provided new tools for early diagnosis and prognosis. For instance, a study employing weighted gene co-expression network analysis (WGCNA) and machine learning models, such as random forest and support vector machines, demonstrated exceptional accuracy (99.81%) in diagnosing colon cancer and its stages. The integration of proteomic and genomic data allowed for the identification of key prognostic markers, including GCNT2 and GLDN, which are associated with disease progression and treatment outcomes. This approach underscores the potential of leveraging AI-driven models to enhance cancer surveillance in high-risk IBD patients, facilitating timely interventions and personalized treatment strategies [124].

Similarly, advancements in AI-based methodologies are transforming the evaluation of UC. A novel deep learning framework, UCFN-Net, has demonstrated superior performance in grading endoscopic images of UC, achieving an accuracy of 89.57% on private datasets. By incorporating fine-grained lesion feature learning and noise suppression gating, this model addresses the challenges of small lesion detection and feature loss during transmission. The implementation of such automated tools not only standardizes disease assessment but also supports more precise treatment planning and earlier prevention of complications [125].

Beyond traditional therapeutic targets, molecular advances in IBD are increasingly unlocking new possibilities in personalized medicine. For instance, proteomic studies have revealed early markers of therapeutic response, with oncostatin M, a pro-inflammatory cytokine linked to steroid-resistant IBD, emerging as a promising biomarker for predicting outcomes and optimizing biologic therapies [126]. Genomic research has further advanced understanding by uncovering genetic variations, such as those in TNFSF15, that are associated with disease severity and therapeutic resistance, offering new opportunities for personalized interventions [127]. In parallel, metabolomic analyses have highlighted the critical role of short-chain fatty acids, like butyrate, in maintaining intestinal barrier function, opening avenues for microbiota-based therapies aimed at restoring gut homeostasis [128]. Collectively, these advances emphasize the transformative potential of molecular profiling in routine care, paving the way for precision medicine approaches that enhance treatment effectiveness, reduce complications, and improve long-term quality of life for IBD patients. However, significant challenges, including high costs, data integration complexities, and population variability, continue to hinder the seamless translation of these discoveries into clinical practice.

Building on these molecular insights, recent scientific evidence is exploring the potential of combination advanced therapies for IBD treatment: for instance, a study reveals corticosteroid-free clinical stable response rates at weeks 12 and 52 weeks, highlighting the potential of these combination therapies to address refractory cases, though more considerations regarding safety profiles and financial accessibility are still needed [127,129]. In parallel, patient-centered outcomes are becoming indispensable components of IBD management. Incorporating PROs into clinical practice provides a comprehensive understanding of how IBD affects individuals’ daily lives, covering aspects like symptom burden, quality of life, treatment satisfaction, and functional well-being. By prioritizing PROs, clinicians can not only enhance patient engagement but also tailor treatment decisions more effectively, ultimately fostering personalized and impactful care strategies [130,131].

The future of IBD management lies in a multi-dimensional strategy that integrates clinical, endoscopic, histologic, transmural, and molecular assessments to achieve durable remission while enhancing patient quality of life. The evolution of IBD management has increasingly emphasized not only clinical and endoscopic remission, but also long-term patient-centered outcomes. Recent evidence highlights that achieving deeper treatment targets, such as HH in UC and TH in CD, correlates with improved quality of life and reduced long-term complications, including surgeries and hospitalizations. The STRIDE-II consensus underscores the importance of these targets alongside biomarker normalization and patient-reported outcomes (PROs) to restore daily functioning and minimize disease burden. Furthermore, the SPIRIT consensus advocates for incorporating disease-modification goals that address the broader impacts of IBD, such as disability and cancer prevention, thus reaffirming the role of early, tailored therapeutic strategies in enhancing both immediate and sustained patient well-being [126,132]. Realizing this vision will require addressing the practical challenges of applying these targets, validating their effectiveness across diverse populations, and refining their integration into the T2T framework.

## Figures and Tables

**Table 1:** Current and emerging targets in IBD.

Category	Crohn’s Disease	Ulcerative Colitis
Clinical remission	Defined by CDAI < 150, HBI < 5, or PRO2 score with abdominal pain ≤ 1 and stool frequency ≤ 3	Defined by PRO2 score: rectal bleeding = 0 and stool frequency normalized, or partial Mayo score < 3 (no subscore > 1)
Biomarkers	Normalization of CRP and fecal calprotectin (to 100–250 mg/g) Used for monitoring subclinical inflammation and treatment response	Normalization of CRP and fecal calprotectin (to 100–250 mg/g) FC predicts clinical relapse, sustained response, endoscopic remission, and histologic remission
Endoscopic remission	SES-CD <3 points or absence of ulcerations Associated with reduced hospitalizations, durable remission, and better outcomes	MES ≤ 1; complete remission (MES = 0) Linked to fewer relapses, improved quality of life, and better PROs scores
Histologic remission	Not routinely recommended due to discontinuous inflammation; limited evidence supports histologic remission for better long-term outcomes	An emerging target reflecting deeper remission Associated with reduced relapse and colorectal neoplasia Defined as absence of neutrophils, erosions, and ulcerations per ECCO guidelines
Transmural remission and new IUS targets	Reflects full-thickness resolution of inflammation; Assessed via IUS, MRE, and CT Linked to reduced hospitalizations and improved outcomes compared to mucosal remission	Not a primary target in UC, but imaging techniques (IUS, TPUS) are emerging to assess inflammation and remission MUC > 6.2 correlates with active disease MUC criteria (BWT + CDS) identified as potential treatment targets to predict outcomes
Disease clearance	Not defined as a formal goal in CD	A multidimensional target combining clinical, endoscopic, and histologic remission Associated with improved outcomes, fewer hospitalizations, and lower surgery rates
Emerging molecular targets	Identifying molecular pathways, proteomics, and genetic markers to guide treatment and predict progression	Specific epigenetic and proteomic markers associated with disease severity and risk of colorectal cancer are under investigation
Quality of life	Improvement of health-related quality of life through reduced disability and disease burden	

CDAI: Crohn Disease Activity Index; HBI: Harvey–Bradshaw Index; PRO2: patient-reported outcome; CRP: C-reactive protein; SES-CD: Simple Endoscopic Score for Crohn’s Disease; MES: Mayo Endoscopic Subscore; IUS: intestinal ultrasound; MRE: magnetic resonance enterography; CT: computed tomography; TPUS: transperineal ultrasound; MUC: Milano Ultrasound Criteria; BWT: bowel wall thickness; CDS: color Doppler signal; CD: Crohn’s disease.

## Data Availability

No new data were created or analyzed in this study. Data sharing is not applicable to this article.
